# Supplementary material for: Development and validation of FootNet; a new kinematic algorithm to improve foot-strike and toe-off detection in treadmill running
Source: PLoS One. 2021 Aug 9;16(8):e0248608. doi: 10.1371/journal.pone.0248608 (PMC8351929; doi:10.1371/journal.pone.0248608)
Supplement: S2 Fig — Top row: mean (± standard deviation) hip, knee and ankle sagittal plane angles during the gait cycle beginning from highest foot COM position to highest foot COM position. Average foot strike and toe off are indicated with vertical dashed lines to aid interpretation. More positive angles refer to dorsiflexion in the ankle plot. The violin plots show the error (°) distribution in hip, knee and ankle sagittal plane angles at foot strike (2nd row) and toe off (4th row) as a function of anticipated (negative time values on the x axis) or delayed (positive time values) step event detection, where the correct value of the variable is taken from the event time provided by the “gold standard” method (force plate). The horizontal black line within each violin represents the mean. Error classification in acceptable (light blue), reasonable (dark blue) and unacceptable (red) are displayed for foot strike (third row) and toe off (fifth row) respectively. (DOCX) [file pone.0248608.s002.docx]

| **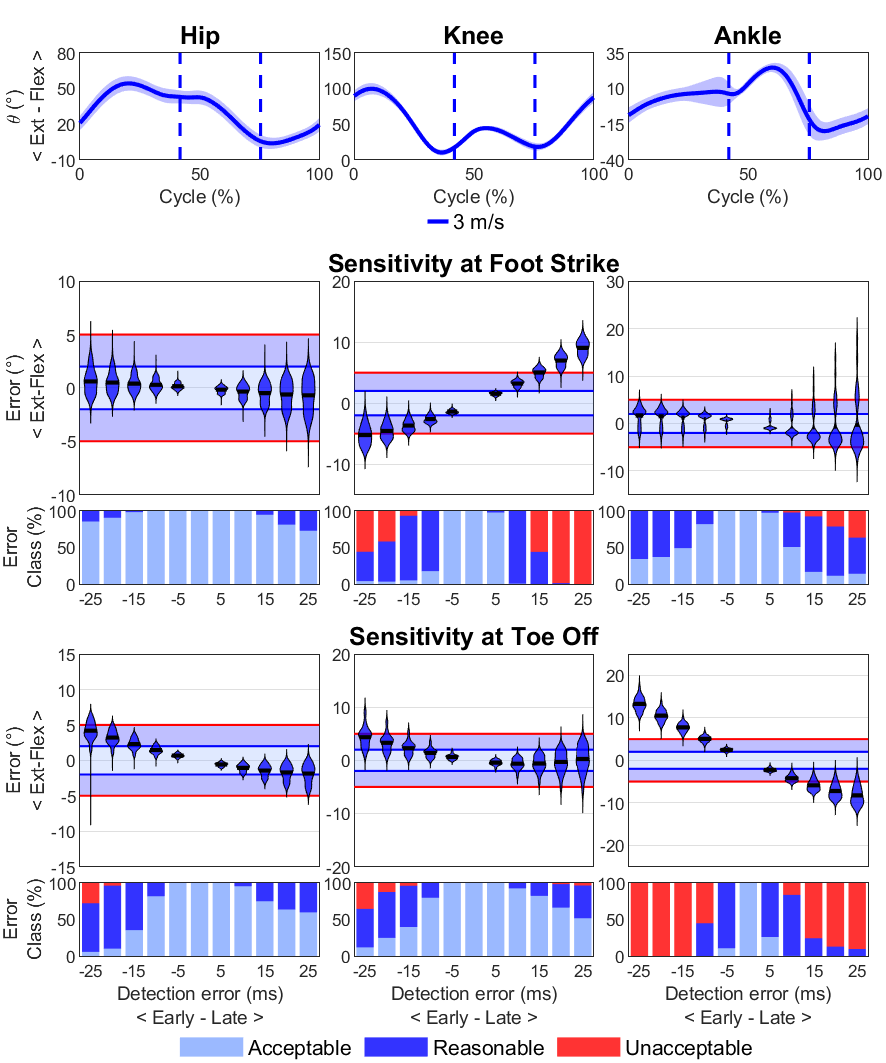** |
| --- |
| Sensitivity analysis. Top row: mean (± standard deviation) hip, knee and ankle sagittal plane angles at 3 m/s during the gait cycle beginning from highest foot COM position to highest foot COM position. Average foot strike and toe off are indicated with vertical dashed lines to aid interpretation. More positive angles refer to dorsiflexion in the ankle plot. The violin plots show the error (°) distribution in hip, knee and ankle sagittal plane angles at foot strike (2^nd^ row) and toe off (4^th^ row) as a function of anticipated (negative time values on the x axis) or delayed (positive time values) step event detection, where the correct value of the variable is taken from the event time provided by the “gold standard” method (force plate). The horizontal black line within each violin represents the mean. Error classification in acceptable (light blue), reasonable (dark blue) and unacceptable (red) are displayed for foot strike (third row) and toe off (fifth row) respectively. |
